# A comprehensive appraisal of mechanism of anti-CRISPR proteins: an advanced genome editor to amend the CRISPR gene editing

**DOI:** 10.3389/fpls.2023.1164461

**Published:** 2023-06-23

**Authors:** Nisha Choudhary, Dipty Tandi, Rakesh Kumar Verma, Virendra Kumar Yadav, Naveen Dhingra, Tathagata Ghosh, Mahima Choudhary, Rajarshi K. Gaur, Magda H. Abdellatif, Amel Gacem, Lienda Bashier Eltayeb, Mohammed S. Alqahtani, Krishna Kumar Yadav, Byong-Hun Jeon

**Affiliations:** ^1^ Department of Biosciences, School of Liberal Arts and Sciences, Mody University of Science and Technology, Lakshmangarh, Rajasthan, India; ^2^ Department of Agriculture, Medi-Caps University, Indore, Madhya Pradesh, India; ^3^ Department of Arts, School of Liberal Arts and Sciences, Mody University of Science and Technology, Lakshmangarh, Rajasthan, India; ^4^ Department of Biotechnology, Deen Dayal Upadhyaya (D.D.U.) Gorakhpur University, Gorakhpur, Uttar Pradesh, India; ^5^ Department of Chemistry, College of Sciences, Taif University, Taif, Saudi Arabia; ^6^ Department of Physics, Faculty of Sciences, University 20 Août 1955, Skikda, Algeria; ^7^ Department of Medical Laboratory Sciences, College of Applied Medical Sciences, Prince Sattam Bin AbdulAziz University-Al-Kharj, Riyadh, Saudi Arabia; ^8^ Radiological Sciences Department, College of Applied Medical Sciences, King Khalid University, Abha, Saudi Arabia; ^9^ Research Center for Advanced Materials Sciences (RCAMS), King Khalid University, Abha, Saudi Arabia; ^10^ Faculty of Science and Technology, Madhyanchal Professional University, Ratibad, India; ^11^ Environmental and Atmospheric Sciences Research Group, Scientific Research Center, Al-Ayen University, Thi-Qar, Nasiriyah, Iraq; ^12^ Department of Earth Resources and Environmental Engineering, Hanyang University, Seoul, Republic of Korea

**Keywords:** anti-CRISPR, genome editing, Acr inhibitors, genetic memory, CRISPR-Cas complex, DNA binding inhibition, DNA cleavage

## Abstract

The development of precise and controlled CRISPR-Cas tools has been made possible by the discovery of protein inhibitors of CRISPR-Cas systems, called anti-CRISPRs (Acrs). The Acr protein has the ability to control off-targeted mutations and impede Cas protein–editing operations. Acr can help with selective breeding, which could help plants and animals improve their valuable features. In this review, the Acr protein–based inhibitory mechanisms that have been adopted by several Acrs, such as (a) the interruption of CRISPR-Cas complex assembly, (b) interference with target DNA binding, (c) blocking of target DNA/RNA cleavage, and (d) enzymatic modification or degradation of signalling molecules, were discussed. In addition, this review emphasizes the applications of Acr proteins in the plant research.

## 1 Introduction

Bacteria and their viruses (phage) have been engaged in a never-ending weapon race for over 3 billion years. Bacteria are a human microbiome component, making them more intricately linked to people than other organisms. Numerous bacteria are able to coexist with people without causing harm due to the establishment of mutualistic relationships; nevertheless, some of these bacteria are pathogenic (Berg et al., 2020). Bacteriophages, on the other hand, are ubiquitous in the microbial world. There are currently more than a thousand phages that attack a bacterium each and every day (Knott and Doudna, 2018). Restriction modification, superinfection exclusion, an abortive infection mechanism, and other activities make up the anti-phage defensive system, which helps bacteria fend off viruses and other invaders. That is all part of the innate immunity of the bacterial defense system. Bacteria also contain the CRISPR-Cas system, an adaptive immunological defense mechanism (Abedon, 2012). CRISPR-Cas is a self- and non-discriminatory system that is found in Archaea and Bacteria. It is responsible for the development of an adaptive defense mechanism in these organisms by combining highly specialized nucleases with genetic memory (Eitzinger et al., 2020). CRISPR is named after the sequence’s actual nature, which is “clustered regularly interspaced short palindromic repeats”. Prokaryotes use CRISPR and its associated protein (Cas9) as part of their adaptive immune response to viruses and bacteriophages (Hille and Charpentier, 2016).

The CRISPR defensive system consists of three fundamental stages: adaption (spacer acquisition), crRNA synthesis (expression), and target interference. These stages work together to protect bacteria from multiple viral attacks (Hille and Charpentier, 2016). CRISPR loci are clusters of regularly interspaced short repeats that can be found in the chromosomal or plasmid DNA of certain prokaryotes. The Cas gene, which encodes the nuclease protein (Cas protein) responsible for destroying or cleaving viral nucleic acid, is typically located next to the CRISPR gene (Asmamaw and Zawdie, 2021). The Cas proteins detect the foreign invading DNA, cut it up, insert it into the CRISPR spacer area, and store the fragments in the genome (adaptation). The transcription of the CRISPR region results in the production of pre-crRNA, which is then processed into smaller RNA units called CRISPR RNA (crRNA) (expression). crRNA’s spacer sequence homology aids in the capture of foreign DNA, which is then cleaved by a complex containing the nuclease-active Cas protein (Interference) (Sontheimer and Davidson, 2017; Ishino et al., 2018). Because of their role, crRNAs are also known as guide RNAs (gRNAs) (Brouns et al., 2008). Short viral DNA fragments (spacers) are inserted into the CRISPR array of a bacterial cell during the adaptation process, immunizing the cell against the virus. Thus, spacers act as a genetic memory of earlier viral infections. This is how the CRISPR region remembers previously invaded bacteriophages or viruses and passes down their molecular memory from generation to generation (Ran et al., 2013).

The Red Queen hypothesis postulates that, in order for species to avoid extinction, they will need to continually evolve new mechanisms of resistance to parasites (Brouns et al., 2008). In response, the parasites develop their own countermeasures, which allow them to evade the resistance mechanisms. This battle for survival can be traced back to the co-evolutionary dynamics of bacterial populations and bacteriophages (Pawluk et al., 2018). Through counter defense evolution in the microbial world, phages have evolved the inhibitors of CRISPR-Cas systems. The first known CRISPR-Cas inhibitors were found in a family of phages from the genus *Pseudomonas* spp. Although they had protospacer sequences that should have been targeted by this system, these phages were, nonetheless, able to infect and multiply in a *Pseudomonas aeruginosa* strain with an active type I F CRISPR-Cas system (Bondy-Denomy et al., 2013). Phages encode anti-CRISPR (Acr) proteins to evade the CRISPR-Cas immune system of their hosts (Maxwell, 2017). Acr proteins invade viral DNA into bacteria, shielding the phage’s genetic material from the CRISPR system (Semenova et al., 2011). Acr proteins have been studied structurally and biochemically, and their inhibitory effects have been found to encompass a broad array, from blocking crRNA loading to preventing target DNA recognition and DNA cleavage (Davidson et al., 2020; Pinilla-Redondo et al., 2020). To date, there have been 98 unique families of Acr proteins described against CRISPR-Cas systems. The search for new Acr has been aided by the development of bioinformatics tools such as AcRanker, AcrFinder, and PaCRISPR and online databases like Anti-CRISPRdb, AcrDB, AcrHub, and AcrCatalog (Bowen et al., 2022).

CRISPR-Cas is being used for genome engineering in plants and programmable gene regulation, highlighting the necessity for control mechanisms for its various activities (Calvache et al., 2022). Acr proteins, in this light, are an underutilized regulatory mechanism in plant biotechnology. In both herbaceous and woody plant species, Acr proteins are effective at inhibiting CRISPR-Cas9–based genome-editing tools (Liu et al., 2023). In this review, the numerous types of Acr proteins, their respective mechanisms of action, and the broad range of applications were discussed. In contrast to earlier studies, this review includes the most recent data on Acr proteins, their uses, and a detailed explanation of their mechanisms of action.

## 2 Genome editing with CRISPR-Cas

CRISPR-Cas is a revolutionary targeted genome-editing approach that can modify a genome from any region of any species with great precision and accuracy without causing harm to other genes (Koonin and Makarova, 2019). CRISPR loci serve as a genetic library, consisting of two parts: the first is the CRISPR array, which represents the immunological memory primarily derived from foreign genetic elements encoded within individual spacers separated by a gap (Koonin and Makarova, 2019) and the second is crRNA, coupled with one or more Cas proteins in the resulting CRISPR-cas complex, which identifies the invading foreign genetic material. The protospacer, which is frequently flanked by a protospacer neighboring motif, is the location of invading DNA targeted by the spacer [proto-spacer adjacent motif (PAM), usually 2–4 nucleotides]. PAM sequences are important in most circumstances because they help the cell to distinguish between its own DNA and invading foreign DNA. For instance, when foreign DNA gets in the proximity of crRNA, it forms the complex with Cas protein followed by cleavage and destruction of foreign DNA (Zhang, 2019). The CRISPR-Cas system is highly diverse, and, based on the complement of unique Cas genes, length of spacers, and difference in palindromic repeats, the CRISPR-Cas system is classified into two classes: six subclasses (types I–VI) and numerous sub-subclasses (subtypes). The CRISPR-Cas system is divided into two classes based on the functions and structure of Cas-protein (CRISPR associate protein) (Makarova et al., 2015; Mohanraju et al., 2016; Zhang, 2019). The class 1 systems include a multi-subunit Cas protein complex (types I, III, and IV) to recognize and cleave the nucleic acid. The class 2 system (types II, V, and VI) includes large and multifunctional Cas proteins, for example, Cas9 that participates in DNA targeting and cleavage (Zhang, 2019). In recent years, the development of CRISPR-based genome-editing technologies has revolutionized the fields of molecular biology and genetics (Maxwell, 2017). CRISPR-Cas technology, as a genome-editing tool, is tremendously useful in preventing genetic disease and fighting genetic abnormalities (Maxwell, 2017). Furthermore, by generating genomes that are more environmentally friendly, the CRISPR-Cas technique is being used to improve the quality of valuable commercial plants and to find solutions for a number of hereditary disorders.

## 3 Anti-CRISPR proteins

Bacteria use the most dependable immune system, CRISPR-Cas9, to defend themselves against phage attacks. Prior to the discovery of the Acr protein, the only option for phages to avoid CRISPR-Cas–mediated destruction was by point mutations (Loureiro and Da Silva, 2019). Changes in their sequences make bacteria unable to recognize the phage genome, allowing phages to live for longer periods of time. However, they are only protected until the bacteria reproduce their new sequence with mRNA and insert it into the CRISPR area. Acr proteins, which block the CRISPR-Cas system, have evolved in phages to circumvent CRISPR-Cas–mediated immunity (Maxwell, 2017). Infection with a CRISPR-targeted phage in the presence of *P. aeruginosa* prophage was monitored using a functional test (Jooyoung et al., 2018), Bioinformatic linkages with known aca genes and Acr proteins “guilt by association”, self-targeting within the same genome, were used in bioinformatic investigations to identify latent Acr candidates in prophages. The lytic phage is being screened for those who do not receive an immune response from the CRISPR system (Maxwell, 2017).

Acrs are bacteriophage genes that were formerly recognized as auxiliary genes (Juhala et al., 2000). Although these accessory genes are not required for the phage’s life cycle, their presence among phages suggests that they provide an evolutionary benefit in some circumstances (Harald et al., 2004). Recently, it was discovered that the CRISPR-Cas defensive system is only present in 50% of bacteria, in which case, Acr proteins are inappropriate and apparent Acr proteins are absent. Acr proteins are also known as inhibitor proteins because they stop the CRISPR-Cas system from destroying the phage genome. In 2013, the first Acr protein was discovered in a collection of closely related phages that infect *Pseudomonas aeruginosa* (Bondy-Denomy and Davidson, 2014). They permitted a phage that should have been targeted and destroyed by the type I-F CRISPR-Cas system to infect and kill the bacterium after integrating these phages into the bacterial genome (a form known as prophage). In *P. aeruginosa*, four additional Acr proteins (Acr IE1–4) were discovered to block the I-E CRISPR-Cas system (Jooyoung et al., 2018). As a result of applying the guilt-by-association strategy, the first inhibitors of the type II CRISPR-Cas system were found. Three Acr protein families—AcrIIC1, AcrIIC2, and AcrIIC3—were found to block the activity of type II-C CRISPR-Cas9 in *Neisseria meningitides* and were the first Acr proteins used for Cas9-mediated genome editing in human cells (Pawluk et al., 2016a). Pawluk and their team used a lytic phage technique to identify AcrIIA5 and AcrIIA6 proteins in two virulent phages, and AcrIIA5 has proven to be the most broad-spectrum inhibitor of the type II CRISPR-Cas proteins. Although a large number of Acr proteins have been found (**Table 1**), only a handful have been thoroughly described until now. AcrIIA1, AcrIIA4, AcrFI, AcrFII, AcrF3, and AcrF10 are the most studied Acrs.

**Table 1:** Types of anti-CRISPR proteins and their known mechanism of action.

Family of anti-CRISPR protein	Source of anti-CRISPR	Size of amino acid	Type of CRISPR inhibited	Known mechanism of action	References
AcrIC1	*Moraxella bovoculi* prophage	190	I-C (Pae, Mbo)		(Marino et al., 2018; Zhang, 2019)
AcrIC3	*P. aeruginosa*			Binds to Cas3 or cascade in a way that prevents Cas3 recruitment or DNA cleavage, while allowing cascade-DNA binding	(León et al., 2021)
AcrIC4	*P. aeruginosa*			Blocked CRISPRi	(León et al., 2021)
AcrIC5	*P. aeruginosa*			Blocked CRISPRi	(León et al., 2021)
AcrIC6	*P. aeruginosa*			Did not block CRISPRi but given its weak activity	(León et al., 2021)
AcrIC7	*P. stutzeri*			Blocked CRISPRi	(León et al., 2021)
AcrIC8	*P. aeruginosa*			Blocked CRISPRi	(León et al., 2021)
AcrID1	*Sulfolobus islandicus rudivirus 3*	98	I-D (Sis)	DNA binding, binds as a dimer to the Cas10d mimicking DNA Possible allosteric inhibition by inducing cascade-crRNA dimerization	(He et al., 2018; Bhoobalan-Chitty et al., 2019; Yu and Marchisio, 2020; Jia and Patel, 2021)
AcrIE1	*Pseudomonas aeruginosa* phage JBD5	100	I-E (Pae)	DNA cleavage, binds as a dimer to the Cas3	(April et al., 2014; Bondy-Denomy et al., 2015)
	*P. aeruginosa* phage JBD88a	84	I-E (Pae)		(Bondy-Denomy et al., 2015)
AcrIE3	*P. aeruginosa* phage DMS3	68	I-E (Pae)	Probably binds to the cascade (blocks DNA binding)	(April et al., 2014; Bondy-Denomy et al., 2015)
AcrIE4	*P. aeruginosa* phage D3112	52	I-E (Pae)		(Bondy-Denomy et al., 2015)
AcrIE5	*Pseudomonas otitidis* prophage		I-E (Pae)		(Zhang, 2019)
AcrIE6	*P. aeruginosa* prophage		I-E (Pae)		(Marino et al., 2018; Zhang, 2019)
AcrIE7	*P. aeruginosa* prophage		I-E (Pae)		(Marino et al., 2018; Zhang, 2019)
AcrIF1	*P. aeruginosa* phage JBD30	78	I-F (Pae, Pec)	Blocks DNA binding, two to three copies interact with the hexameric Cas7f spine of the cascade Blocking crRNA-DNA hybridization	(Chowdhury et al., 2017; Dong et al., 2017; Peng et al., 2017; Yu and Marchisio, 2020; Yu and Marchisio, 2020)
AcrIF2	*P. aeruginosa* phage D3112	90	I-F (Pae, Pec)	Blocks DNA binding, binds to the Cas5f: Cas8f tail of the cascade, mimicking DNA Mimicking the negative charge on DNA and disrupting the interaction between the crRNA phosphate backbone and the Csy complex	(Chowdhury et al., 2017; Dong et al., 2017; Peng et al., 2017; Yu and Marchisio, 2020)
AcrIF2	*P. aeruginosa* phage D3112	90	I-F (Pae, Pec)	Inhibits DNA binding by partially overlapping with the binding site of dsDNA	(Westra et al., 2012; Pawluk et al., 2016a)
AcrIF3	*P. aeruginosa* prophage JBD5	139	I-F (Pae)	Prevents cas3 recruitment by cascade and blocks the entrance of the DNA binding tunnel; blocks new sequence attainment with the active site within the RuvC domain; hinders the conformational change of the HNH domain Binding Cas3 by mimicking the helical bundle of Cas8f	(Westra et al., 2012; April et al., 2014; Pawluk et al., 2016a; Pawluk et al., 2016a; Pawluk et al., 2016b; Dong et al., 2017; Yu and Marchisio, 2020)
AcrIF4	*P. aeruginosa* phage JBD26	100	I-F (Pae)	Blocks DNA binding, binds to the cascade	(Bondy-Denomy and Davidson, 2014)
AcrIF5	*P. aeruginosa* phage JBD5	79	I-F (Pae)		(Bondy-Denomy et al., 2015)
AcrIF6	*P. aeruginosa* prophage	100	I-F (Pae, Pec), I-E [Pae]	Binds at the junction between Cas7.6f and Cas8f to inhibit DNA duplex splitting Interaction with Cas8f (at K247) that prevents DNA opening for crRNA-DNA hybridization	(Pawluk et al., 2016a; Jooyoung et al., 2018; Yu and Marchisio, 2020)
AcrIF7	*P. aeruginosa* prophage	83	I-F (Pae, Pec)		(Pawluk et al., 2016b)
AcrIF8	*Pectobacterium carotovorum* phage ZF40	92	I-F (Pae, Pec)	Binds to the Csy spiral backbone to prevent DNA hybridization Interaction with crRNA that disrupts crRNA hybridization with DNA and prevents crRNA-DNA heteroduplex propagation	(Pawluk et al., 2016a; Pawluk et al., 2016a; Pawluk et al., 2016b; Jooyoung et al., 2018; Yu and Marchisio, 2020)
AcrIF9	*Vibrio parahaemolyticus* mobile genetic element	68	I-F (Pae, Pec)	Binds to the Csy spiral backbone to prevent DNA binding Competition with DNA for the lysines in Cas7f subunits that are responsible for DNA binding	(Pawluk et al., 2016a; Jooyoung et al., 2018; Yu and Marchisio, 2020)
AcrIF10	*Shewanella xiamenensis* prophage	97	I-F (Pae, Pec)	DNA mimic, blocks DNA binding Competition with DNA (via DNA mimic) for binding Cas5f-Cas8f	(Pawluk et al., 2016a; Pawluk et al., 2016a; Pawluk et al., 2016b; Jooyoung et al., 2018; Yu and Marchisio, 2020)
AcrIF11	*P. aeruginosa* mobile genetic element	132	I-F (Pae)	Mediated ADP-ribosylation of the Csy complex prevents dsDNA binding	(Marino et al., 2018)
AcrIF12	*P. aeruginosa*	124	I-F (Pae)		(Marino et al., 2018)
AcrIF13	*Moraxella catarrhalis* prophage	115	I-F (Mbo)		(Marino et al., 2018)
AcrIF14	*M. catarrhalis* phage Mcat5	124	I-F (Mbo)	Blocking hybridization of target DNA with the crRNA guide	(Jia and Patel, 2021)
AcrIF15			I–F	Target DNA binding	(Pinilla-Redondo et al., 2020)
AcrIF16					(Pinilla-Redondo et al., 2020)
AcrIF17	*Citrobacter* sp.				(Pinilla-Redondo et al., 2020)
AcrIF18			I–E; I–F	Target DNA binding	(Pinilla-Redondo et al., 2020)
AcrIF19					(Pinilla-Redondo et al., 2020)
AcrIF20					(Pinilla-Redondo et al., 2020)
AcrIF21					(Pinilla-Redondo et al., 2020)
AcrIF22			I–E; I–F		(Pinilla-Redondo et al., 2020)
AcrIF23					(Pinilla-Redondo et al., 2020)
AcrIF24	*Pseudomonas aeruginosa*		I-F	Bound to type I-F cascade, specifically to Cas7 *via* its head domain	(Kim et al., 2022)
AcrIIA1	*Listeria monocytogenes* prophage J0161	149	II-A (Lmo)	Recognizes nucleic acids (putative transcriptional regulation)	(Rauch et al., 2017)
AcrIIA2	*L. monocytogenes* prophage J0161a	123	II-A (Lmo, Spy)	Binds to the PAM-interacting, the WED, the HNH, and the REC2 domains (blocks DNA recognition, binding/unwinding, and cleavage, respectively)	(Rauch et al., 2017; Liu et al., 2018)
AcrIIA3	*L. monocytogenes* prophage SLCC2482	125	II-A (Lmo)		(Rauch et al., 2017)
AcrIIA4	*L. monocytogenes* prophage J0161b	87	II-A (Lmo, Spy)	Binds to the PAM-interacting, the Topo-homology, and the RuvC domains (blocks DNA recognition, binding/unwinding, and cleavage, respectively)	(Dong et al., 2017; Rauch et al., 2017; Yang and Patel, 2017)
AcrIIA5	*Streptococcus thermophilus* (virulent) phage D4276	140	II-A (Sth1, Sth3, Spy)	Inhibits diverse Cas9 orthologs from type II-A, II-B, and II-C, prevents Cas9 from DNA cleavage without blocking DNA binding, can trap the DNA-bound Cas9 complex, inhibits the activity of RuvC domain of Cas9 independent of HNH domain	(Hynes et al., 2017)
AcrIIA6	*S. thermophilus* (virulent) phage D1811	183	II-A (Sth1)	DNA binding, induces St1Cas9 dimerization	(Hynes et al., 2018; Fuchsbauer et al., 2019; S and Tanuj, 2020)
AcrIIA7	*Metagenomic libraries from human gut*	103	II-A (Spy)		(Uribe et al., 2021)
AcrIIA8	Human gut metagenomic libraries	105	II-A (Spy)		(Uribe et al., 2021)
AcrIIA9	Human gut metagenomic libraries	141	II-A (Spy)		(Uribe et al., 2021)
AcrIIA10	Soil metagenomic libraries	109	II-A		(Uribe et al., 2021)
AcrIIA11	*Clostridium* sp. from human gut metagenome	182	II-A	DNA cleavage	(Forsberg et al., 2019)
AcrIIA12	*Listeria monocytogenes* prophage	83	II-A	DNA binding	(Osuna et al., 2020; S and Tanuj, 2020)
AcrIIA13	*Staphylococcus schleiferi* prophage	128	II-A	DNA cleavage	(S and Tanuj, 2020; Watters et al., 2020)
AcrIIA14	*Staphylococcus simulans* prophage	159	II-A	DNA cleavage	(S and Tanuj, 2020) (Watters et al., 2020)
AcrIIA15	*Staphylococcus delphini* prophage	170	II-A	RNA loading	(S and Tanuj, 2020; Watters et al., 2020)
AcrIIA16	*Listeria monocytogenes* Plasmid	202	II-A	DNA cleavage	(S and Tanuj, 2020)
AcrIIA17	*Enterococcus faecalis* Plasmid	109	II-A	DNA cleavage	(S and Tanuj, 2020)
AcrIIA18	*Streptococcus macedonicus* prophage	182	II-A	DNA cleavage	(S and Tanuj, 2020)
AcrIIA19	*Staphylococcus simulans* Plasmid	124	II-A	DNA cleavage	(S and Tanuj, 2020)
AcrIIA20	*Streptococcus iniae* Prophage	62	II-A		(Eitzinger et al., 2020)
AcrIIA21	*Streptococcus agalactiae prophage*	108	II-A		(Eitzinger et al., 2020)
AcrIIC1	*Neisseria meningitides*	85	II-C (Nme, Cje, Geo, Hpa, Smu)	Binds the HNH domain, shields the catalytic center	(Harrington et al., 2017; Zhang, 2019)
AcrIIC2	*N. meningitides* prophage	123	II-C (Nme, Hpa, Smu)	Blocks DNA binding, binds to the bridge helix (BH)-REC1 region	(Mir et al., 2018; Zhu et al., 2019)
AcrIIC3	*N. meningitides* prophage	116	II-C (Nme, Hpa, Smu)	Induces cas9 dimerization, inhibits DNA binding	(Mir et al., 2018; Zhang, 2019)
AcrIIC4	*Haemophilusparainfluenzea* prophage	88	II-C (Nme, Hpa, Smu	Binds to the Cas9 (blocks DNA binding)	(Mir et al., 2018)
AcrIIC5	*Simonsiella muelleri* transfer element	130	II-C (Nme, Hpa, Smu)	Binds to the Cas9 (blocks DNA binding)	(Mir et al., 2018)
AcrIII-1	*Sulfolobus islandicus* and others with type III sys.			Degradation of cA4	(Athukoralage et al., 2020; S and Tanuj, 2020)
AcrIIIB1	*Sulfolobus islandicus rudivirus* 2		III-B	Csx1 RNase interference	(Bhoobalan-Chitty et al., 2019; S and Tanuj, 2020)
AcrVA1	*Moraxella bovoculi* prophage	170	V-A	DNA binding (1) Interaction with WED and PI domains, PAM sequence mimic (2) (2) Truncation of crRNA in a Cas12a-dependent way	(S and Tanuj, 2020; Yu and Marchisio, 2020)
AcrVA2	*M. bovoculi* prophage	322	V-A		(Marino et al., 2018; S and Tanuj, 2020; Yu and Marchisio, 2020)
AcrVA3	*M. bovoculi* prophage	168	V-A		(Marino et al., 2018; S and Tanuj, 2020)
AcrVA4	*M. bovoculi* mobile element		V-A	DNA binding (1) Inhibition of Cas12a conformational changes required for catalytic activity Dislodging Cas12a-crRNA from DNA (2) (3) Binding to Cas12acrRNA-truncated-DNA complex to decrease the recycle of Cas12a.	(Watters et al., 2018; S and Tanuj, 2020; Yu and Marchisio, 2020)
AcrVA5	*M. bovoculi* mobile element		V-A	DNA binding Permanent inactivation of Cas12a *via* covalent modification (acetyltransferase activity)	(Watters et al., 2018; S and Tanuj, 2020; Yu and Marchisio, 2020)
AcrVIA1	*Leptotrichia wadei* F0279 prophage		VI-A	Inhibits Cas13a RNA targeting	(Lin et al., 2020; S and Tanuj, 2020)
AcrVIA2	*Leptotrichia wadei* F0279 prophage		VI-A	Inhibits Cas13a RNA targeting	(Lin et al., 2020; S and Tanuj, 2020)
AcrVIA3	*Leptotrichia wadei* F0279 prophage		VI-A	Inhibits Cas13a RNA targeting	(Lin et al., 2020; S and Tanuj, 2020)
AcrVIA4	*Leptotrichia wadei* F0279 prophage		VI-A	Inhibits Cas13a RNA targeting	(Lin et al., 2020; S and Tanuj, 2020)
AcrVIA5	*Leptotrichia wadei* F0279 prophage		VI-A	Inhibits Cas13a RNA targeting	(Lin et al., 2020; S and Tanuj, 2020)
AcrVIA6	*Rhodobacter capsulat* R121 prophage		VI-A	Inhibits Cas13a RNA targeting	(Lin et al., 2020; S and Tanuj, 2020)
AcrVIA7	*Leptotrichia buccalis* DSM 1135 prophage		VI-A	Inhibits Cas13a RNA targeting	(Lin et al., 2020; S and Tanuj, 2020)

HNH, an endonuclease domain named for characteristic histidine and asparagine residues; REC2, phosphoacceptor receiver; WED: α/β wedge; PI, PAM Interacting domain; CRISPRI, CRISPR interference.

The CRISPR-Cas9 system has swiftly evolved into a useful tool for improving the genomes of plant species. Plant quality has been enhanced through the manipulation of genes to alter metabolic pathways, to increase stress resistance (Os8N3, OsProDH, OsGS3, and OsNAC45 genes in Rice) (Usman et al., 2021), to increase disease resistance (MdDIPM4 gene in apple; EgIFR gene in Oil palm) (Gan and Ling, 2022), to improve flowering time and plant height (*ZmPHYC1 ZmPHYC2* gene in maize) among other things (Li et al., 2020; Montecillo et al., 2020). Increased oleic acid content, higher amylose content, and a thermo-sensitive genic male sterile line are all results of the CRISPR-Cas9 system’s work with *Oryza sativa* (Sun et al., 2017; Abe et al., 2018; Zhou et al., 2019). The CRISPR-Cas9 system, in a similar manner, confers biotic and abiotic stress resistance or tolerance in plants, for example, including resistance in *Cucumis sativus* to potyviruses such as *Zucchini yellow mosaic virus* and *Papaya ring spot mosaic virus-W* (Chandrasekaran et al., 2016) and immunity in *Cucumis sativus* to *Cucumber vein yellowing virus* (Ipomovirus) infection, drought resistance in *Zea mays* (Shi et al., 2017), and salinity tolerance in rice (Zhang, 2019).

## 4 CRISPR-defeating mechanism

**Figure 1:**
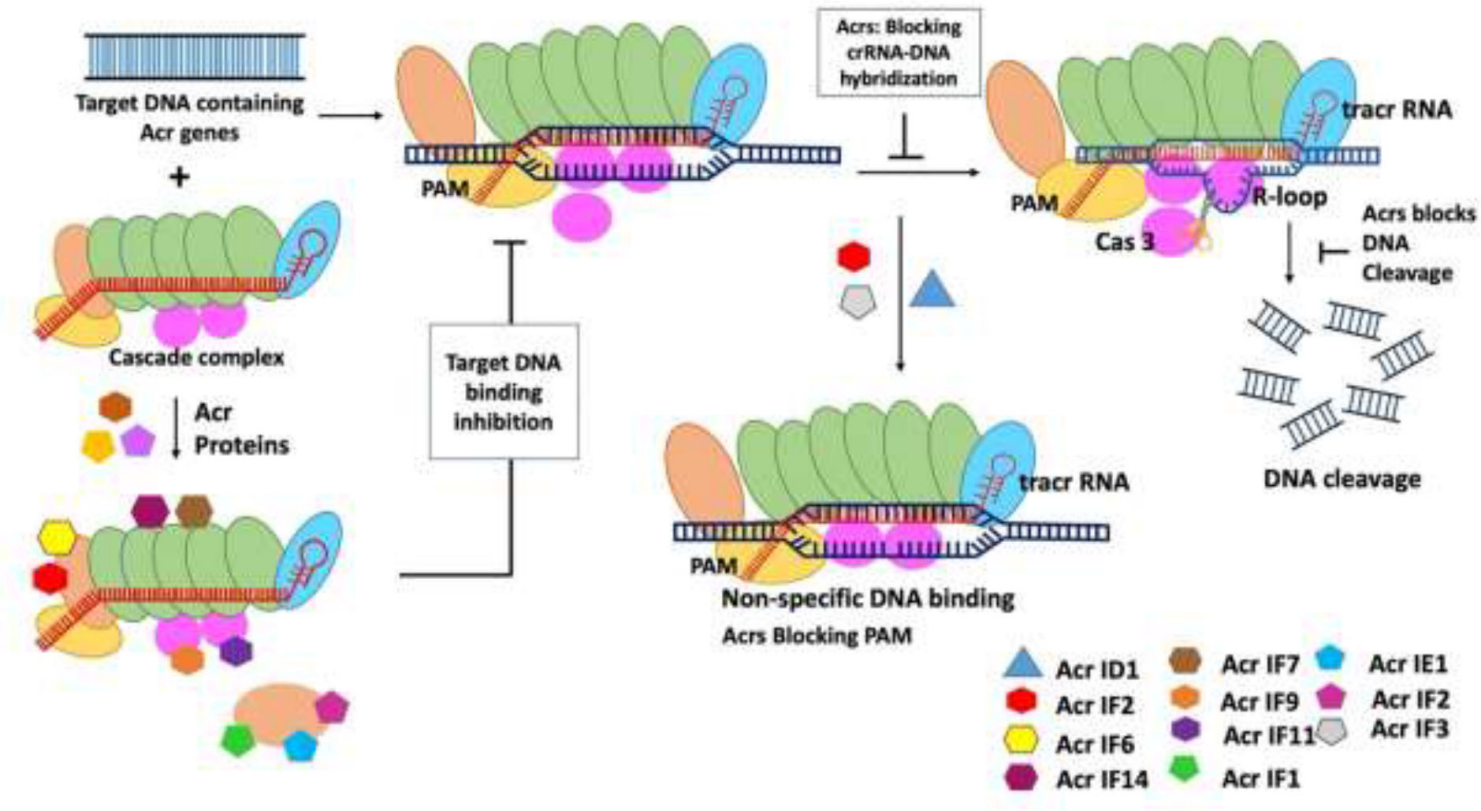
Mechanism of type I anti-CRISPR defense strategy: blocking the crRNA-DNA hybridization, blocking of DNA cleavage, inactivation of Cas3, blocking PAM recognition sites, and causing non-specific dsDNA binding are the main mechanisms adopted by type I anti-CRISPRs. AcrF1, AcrF2, and Acr IE1 attach to the CRISPR complex and prevent it from binding to the DNA target. The non-specific binding of Acr IF2, AcrID1, and AcrIF3 blocks the PAM recognition, and anti-CRISPRs AcrF1–AcrF4 inhibit type 1-F CRISPR complex (Jia and Patel, 2021).

In the long term, it was unknown how phages were able to successfully infect bacteria until Acr proteins were discovered in *Pseudomonas aeruginosa*. Acr proteins can impede the formation of new CRISPR spacers, block cas protein production, obstruct crRNA transcription, prevent the active CRISPR-Cas complex from forming, inhibit binding to foreign DNA elements, and block cleavage activity (Maxwell, 2017). Disruption of DNA binding, inhibition of target sequence, enzymatic degradation of secondary messenger signaling molecule, and cleavage are the four common mechanisms used by Acr proteins (Jia and Patel, 2021). Over the last few years, genetic, biochemical, and structural studies have been used to determine the mechanism of activity of Acr protein families (Pawluk et al., 2016b). Acr proteins do not disrupt the expression of the Cas gene or the process of crRNA but disrupt the DNA binding activity through direct interaction with the CRISPR-Cas complex. In addition, Acr prevents DNA from attaching to the viral genome, allowing it to evade detection (Maxwell, 2017). *P. aeruginosa* type 1-F Acr proteins AcrF1, AcrF2, and AcrF3 have been found to interact directly with the type 1-F CRISPR cascade complex, inhibiting its affinity for DNA binding (**Figure 1**) (Bondy-Denomy et al., 2015). AcrF1 attaches to Cas7F as two to three copies of each monomeric unit in the hexameric unit. Cas7f is more commonly referred to as the type 1-F cascade complex’s backbone (Bondy-Denomy et al., 2015). A key contact is created between a cluster of three essential residues on the surface of AcrF1 and exposed lysine residues in the Cas7F protein backbone, which inhibits DNA targeting access (Bondy-Denomy et al., 2015; Maxwell, 2017). AcrF2 is an acidic protein that acts as a DNA mimic and sterically prevents DNA binding *via* an interaction with the Cas8f-Cas5f heterodimer (Bondy-Denomy et al., 2015). For example, the Acr protein AcrIIA4 binds with the PAM. It is an interacting area of the type 2-A Cas9/Single Guide RNA (sgRNA) complex that keeps the target DNA sequence from getting in the way (Chowdhury et al., 2017). X-ray crystallography and cryo-electron microscopy were utilized to examine these interactions which revealed that AcrF3 binds to the interaction interface, blocking the Cas3 binding site for DNA and locking Cas3 in ADP-bound form. Similarly, type 2-C AcrIIC1 prevents the Cas9 HNH endonuclease from cleaving both target and non-target DNA strains. Potentially, only a few Acr protein structures and mechanisms have been determined to date, and many possible effective strategies may occur with viral Acr gene families (Wang and Wang, 2017).

## 5 Types of anti-CRISPRs

### 5.1 Type I anti-CRISPRs

Researchers have effectively identified type 1 Acr genes (**Table 1**) using various bioinformatics tools (Zhu et al., 2019). The type 1 CRISPR-Cas system is divided into seven subtypes: I-A, IB, I-C, I-D, I-E, I-F, and I-U. In 2013, a disease-causing bacterial species *Pseudomonas aeruginosa* was found to have the first Acr gene encoded by phage. This pathogen had five phage-encoded Acr genes, including AcrF1, which is unique to the type 1-F CRISPR-Cas system in *P. aeruginosa*. Further research revealed that four more Acr genes are present in the same phage operon, namely, AcrE1, AcrE2, AcrE3, and AcrE4, used to block type I-F CRISPR-Cas system of *P. aeruginosa* (Pawluk et al., 2016b). Scientists have been able to identify more Acr genes, such as AcrF6 to AcrF10, in numerous bacterial species encountered by phages in the type 1-F CRISPR system (Pawluk et al., 2016b). AcrF6 is a gene with dual specificity because it can block both type I-F and type I-E CRISPR systems.

Bondy-Donomy and their group reported that the type I Acr proteins are made up of tiny groups of amino acids (about 50–150 amino acids), but there is no sequence similarity between them. Furthermore, the investigators have discovered the mechanism of action of Acr proteins by performing biochemical experiments and introduced four more Acrs AcrF1-AcrF4 *in vitro* and reported that they were able to successfully inhibit type 1-F CRISPR complex (Bondy-Denomy et al., 2015). They also discovered that the CRISPR complex is a 350-crRNA-guided complex composed of 60-nucleotide crRNA and nine Cas proteins including Cas8f and Cas5. These proteins target Cas3, a nuclease helicase, for degradation. A biochemical reaction reveals that AcrF1 and AcrF2 attach to the CRISPR complex and prevent it from binding to the DNA target (Westra et al., 2012). The visualization of structures of Acr and CRISPR binding complex will further improve understanding of the mechanism of Acr and CRISPR complexes interaction as well as inhibition. The main mechanisms of action of type I Acrs are to block crRNA-DNA hybridization to prevent DNA binding, Cas3 inactivation to prevent DNA cleavage, and targeting PAM recognition sites to prevent DNA binding and preventing DNA binding by triggering non-specific dsDNA binding (Jia and Patel, 2021) (**Figure 1**). Recently, Kang and Park (2022) suggested that AcrIC5 may be a DNA mimic Acrs that directly binds to the target DNA binding site in type I-C cascade and inhibits the recruitment of the target DNA to this cascade. This cascade suppresses the recruitment of the target DNA because of this direct binding (Kang and Park, 2022).

### 5.2 Type II anti-CRISPRs

**Figure 2:**
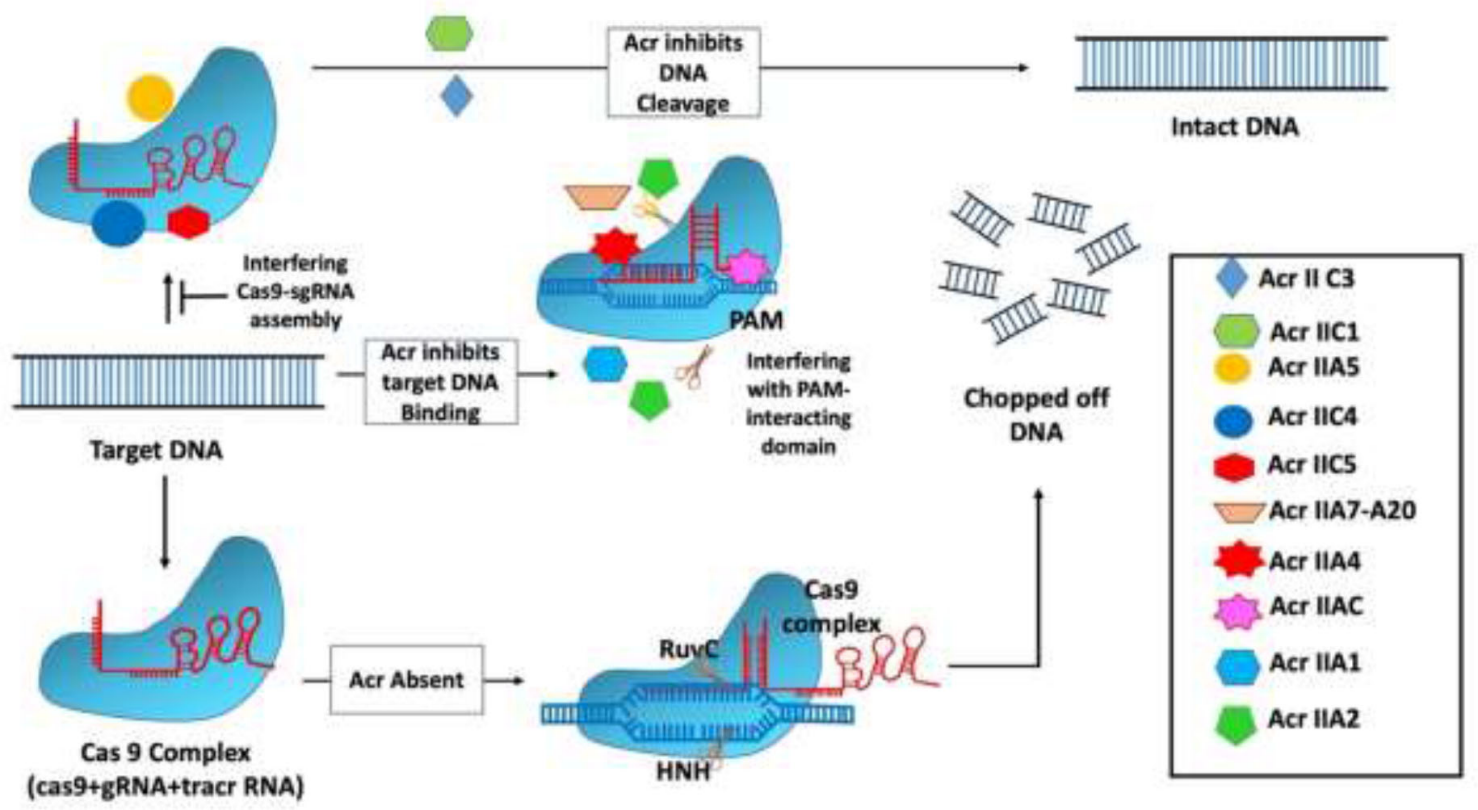
Mechanism of type II anti-CRISPR defense strategy: Type II anti-CRISPRs primarily work by preventing DNA cleavage by direct contact and blocking target DNA binding. Through the binding to catalytic sites in the HNH nuclease domain and the inhibition of the RuvC domain, AcrIIC1 selectively inhibits target DNA cleavage. By binding to the positively charged BH domain, AcrIIC2 blocks the assembly of the surveillance complex, hence blocking guide RNA loading (Jia and Patel, 2021).

The AcrIIC1 protein from *Brackiella oedipodis* was discovered using the bioinformatic technique that was used to uncover type 1 Acrs (Pawluk et al., 2016b). The inhibitory action of AcrIIC1in *Brackiella oedipodis* was found to be quite similar to that of the best-known type 2 CRISPR system (Pawluk et al., 2016b). *Neisseria meningitides* have three robust Acr genes (AcrIIC1, AcrIIC2, and AcrIIC3) that suppress the type 2 CRISPR system. The type 2 CRISPR system in *B. oedipodis* and *N. meningitides* is mostly inhibited by AcrIIC1. Recently, it has been discovered that Acr inhibitors also block the Csy gene by using the self-targeting phenomenon, in which self-targeting is used as a flag in the host genome to indicate the specific gene that needs to be silenced. Following that, many Acr genes, including AcrIIA1, AcrIIA2, AcrIIA3, and AcrIIA4, were identified to suppress the type 2 CRISPR system (**Table 1**) employing BLAST searches with genomic positions similar to monocytogenes prophage (Mir et al., 2018). All of the identified Acrs have the ability to attach to a certain type of CRISPR complex, operate as needed, and follow the necessary process to suppress the bacterial cell’s CRISPR system (**Figure 2**). The main mechanisms of action of type II Acrs are DNA cleavage inhibition by direct interaction and target DNA binding inhibition (Jia and Patel, 2021). Specifically, AcrIIC1 specifically inhibits target DNA cleavage by binding to catalytic sites in the HNH nuclease domain and blocking the RuvC domain (Harrington et al., 2017). AcrIIC2 is responsible for preventing the loading of gRNA because it binds to the positively charged BH domain, which impedes the assembly of the surveillance complex (Sun et al., 2017). In addition, through connecting with the HNH and REC2 domains, two AcrIIC3 proteins bind two Cas9 proteins together. This reduces the mobility of the HNH domain, which, in turn, prevents Cas9 activation. AcrIIC4 exhibited a helical bundle fold consisting of four helices, and it competitively binds to the specific target DNA-binding pocket, which leads to inhibition of Cas9 binding to the target site. In addition, AcrIIC4 attaches to the pocket in such a way that it prevents Cas9 from binding to the pocket (Kim et al., 2021). Likewise, multiple orthologs of type II-C Cas9 enzymes, such as those found in *Neisseria meningitidis* (Nme1Cas9) and *Simonsiella muelleri* (SmuCas9), are inhibited by AcrIIC5 (Sun et al., 2023).

The work by Calvache et al. (2022) demonstrated that two Acr proteins, AcrIIA4 and AcrVA1, function as potent inhibitors of CRISPR-Cas–mediated editing in *N. benthamiana* (Calvache et al., 2022). In addition, Liu and their team have shown that transient expression and stable transformation methods can be used to effectively activate AcrIIA4 and AcrIIA5 in herbaceous and woody plant species. The authors used leaf-infiltration and protoplast-based transient expression to investigate the effects of AcrIIA4 and AcrIA5 activities on the SpCas9-based adenine base editor (ABE7) in the herbaceous plants Arabidopsis (*Arabidopsis thaliana*) and *N. benthamiana*, as well as the woody plant hybrid poplar “717” (*Populus tremula* × *P. alba* hybrid clone INRA 717-1B4), and suggested that both AcrIIA4 and AcrIIA5 are capable of preventing target mutagenesis in the genome of *N. benthamiana* that is mediated by SpCas9/sgRNA (Liu et al., 2023).

### 5.3 Type III anti-CRISPRs

AcrIIIB was discovered from the archaeal virus and is known to inhibit type III-B CRISPR-Cas system (**Table 1**). The mechanism of inhibition is performed by interacting with Cmr effector complexes by AcrIIIB (Bhoobalan-Chitty et al., 2019).

There are three mechanisms of action of type III Acrs as follows:

1. Second-messenger production inhibition: cyclic oligoadenylate (cOA) are cyclic oligoadenylate secondary messengers that are produced in response to the infection of viruses by subtype III-B Cmr complex (Jia and Patel, 2021; Bhoobalan-Chitty et al., 2019; Athukoralage et al., 2019). Second-messenger cOA targeting is one of the strategies used by viruses against eukaryotic cyclic GMP–AMP receptor stimulator of interferon genes (cGAS-STING) innate immunity and prokaryotic CRISPR-Cas immunity.
2. Second-messenger degradation: second-messenger cA4 degrades by direct binding of AcrIII-1 with cOA, resulting in allowing viruses to overcome type III CRISPR-Cas immunity (Athukoralage et al., 2019; Athukoralage et al., 2020; Jia and Patel, 2021).
3. Implications from eukaryotic cGAS-STING immunity (Jia and Patel, 2021).

#### 5.3.1 Virus ring nuclease anti-CRISPRs (role of cyclic nucleotides in defense system)

**Figure 3:**
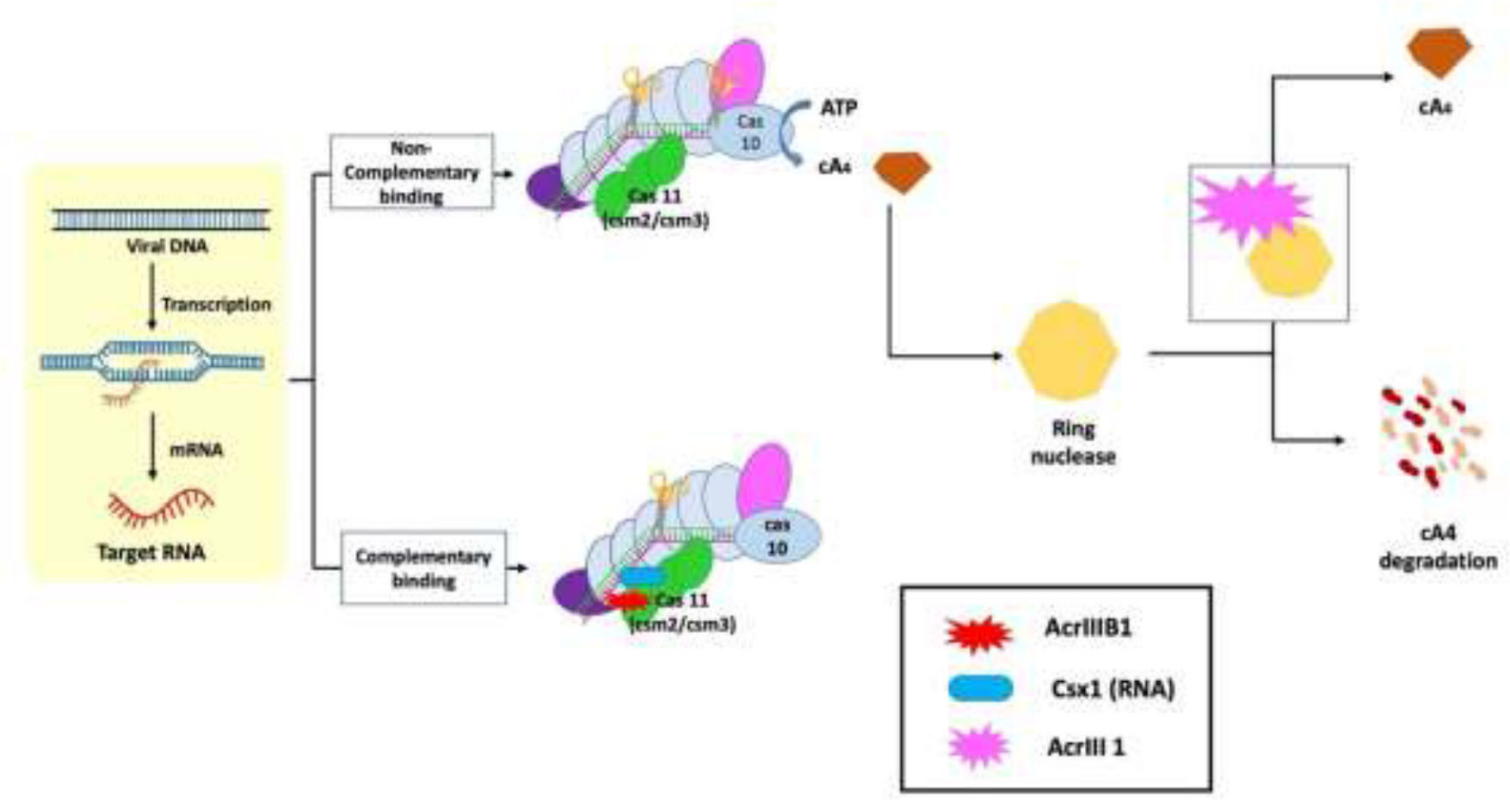
Mechanism of type III anti-CRISPR defense strategy: The main mechanisms of action that are carried out by type III anti-CRISPRs are the destruction of cyclic tetra-adenylate (cA4) and interference with Csx1 RNase. AcrIII-1, a viral ring nuclease, binds to cA4 and blocks the active site necessary to cleave this signalling molecule, effectively silencing the type III CRISPR system (Jia and Patel, 2021).

The molecular actions of AcrIII-1 viral ring nuclease were recently discovered using the type III CRISPR system and viral RNA. When the type III CRISPR system detects viral RNA, it activates two regions of the Cas10 protein: 1) the HD nuclease domain, which degrades viral DNA; and 2) the cyclase domain, which synthesizes cyclic oligoadenylates from ATP. Cyclic nucleotide has become increasingly important in host-pathogen interactions. Finally, researchers discovered a new viral Acr enzyme gene family that rapidly destroys cyclic tetra-adenylate (cA4), a signaling molecule for the bacterial type III CRISPR system. The viral ring nuclease AcrIII-1 binds to cA4 and obstructs the active site to cleave this signaling molecule, allowing the virus to knock out the type III CRISPR system (**Figure 3**). The widespread presence of this Acr in numerous archaeal and bacterial virus families indicates that this enzyme disrupts cellular immunity by cutting down a critical signaling molecule, making it difficult for cells to develop resistance to it. Recent investigations have revealed that bacteria have a variety of cellular defense systems including cyclic nucleotide signaling (Bondy-Denomy et al., 2015; Chowdhury et al., 2017; Maxwell, 2017; Athukoralage et al., 2020). The main mechanisms of action of type III Acrs are the degradation of cA4 and Csx1 RNase interference (Bhoobalan-Chitty et al., 2019; Athukoralage et al., 2020).

### 5.4 Type V anti-CRISPRs

**Figure 4:**
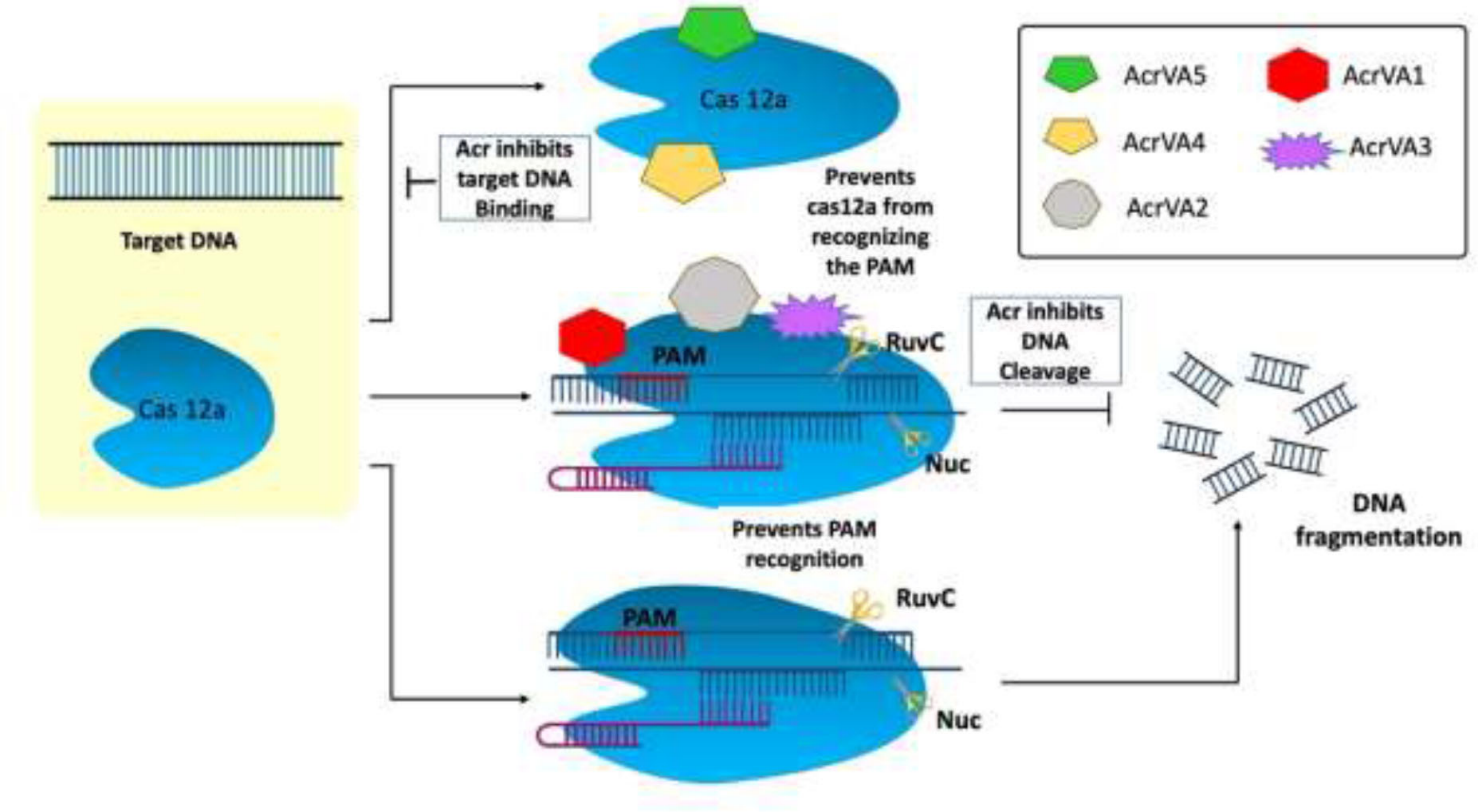
Mechanism of type V anti-CRISPR defense strategy: To cleave crRNA, AcrVA1 performs the role of a DNA mimic and attaches itself to Cas12a in the area of the protein’s PAM-interacting domain. AcrVA1 strongly binds Cas12a central pocket *via* polar interaction and binds with Nuclease domain of C2c1 (NUC) and REC lobe. AcrVA1 occupies salt bridges and hydrogen bonds that interact with PAM. This blocks PAM-Cas12a-crRNA complex DNA cleavage. AcrVA4 binds the Cas12a-crRNA–truncated DNA complex and inactivates it, decreasing Cas12a recycling. AcrVA5, an acetyltransferase, covalently inactivates CRISPR-Cas12a systems (Jia and Patel, 2021).

The type V Acr proteins were exclusively found in subtype A (AcrVA1–AcrVA5). Moraxella bovoculi, a gram-negative bovine pathogen, naturally encodes CRISPR-Cas12a and was the first (and only) to have type V Acrs (acrVA1-5) identified (Marino, 2023). Because *Moraxella bovoculi* has numerous self-targeting sites in its genome, type V Acr and subtypes were discovered in the presence of an AcrIF11 homolog (Marino et al., 2018). AcrVA4 and VA5 are classified differently in *M. bovoculi* (Watters et al., 2018) by Self-Targeting Spacers Search and Cell-Free Transcription-Translation (Marshall and Akbari, 2018). Cas12a has a negatively charged protein called crVA1 that has five helices (1 to 5) and binds to a conserved area on the Cas12a site. This could explain the wide range of AcrVA1 inhibition (**Figure 4**) (Zhang, 2019). AcrVA1 acts as a DNA mimic and attaches to Cas12a in the vicinity of its PAM-interacting domain to cleave crRNA. crRNA is stretched by two helices when AcrVA1 binds to the Cas12a area. Cas12a-mediated DNA cleft efficiency is restored by mutations in the two helices (Bernabé-Orts et al., 2019; Zhang, 2019). Thus, in crRNA-truncation, the two helices exhibit RNase activity. AcrVA2 prevents the production of Cas12a by attaching to conserved regions in the nascent polypeptide of Cas12a and causing the mRNA encoding Cas12a to be degraded (Zhang, 2019). AcrVA4 acts as a surrogate for pre-crRNA, impeding the conformational changes in Cas12a that are necessary for cleavage. AcrVA5 prevents MbCas12a from interacting with PAM, as shown by structural and biochemical analysis. The steric barrier created by acetylation at this location is sufficient to inhibit dsDNA binding and subsequent cleavage (Zhang, 2019; Jia and Patel, 2021).

### 5.5 Type VI anti-CRISPRs

**Figure 5:**
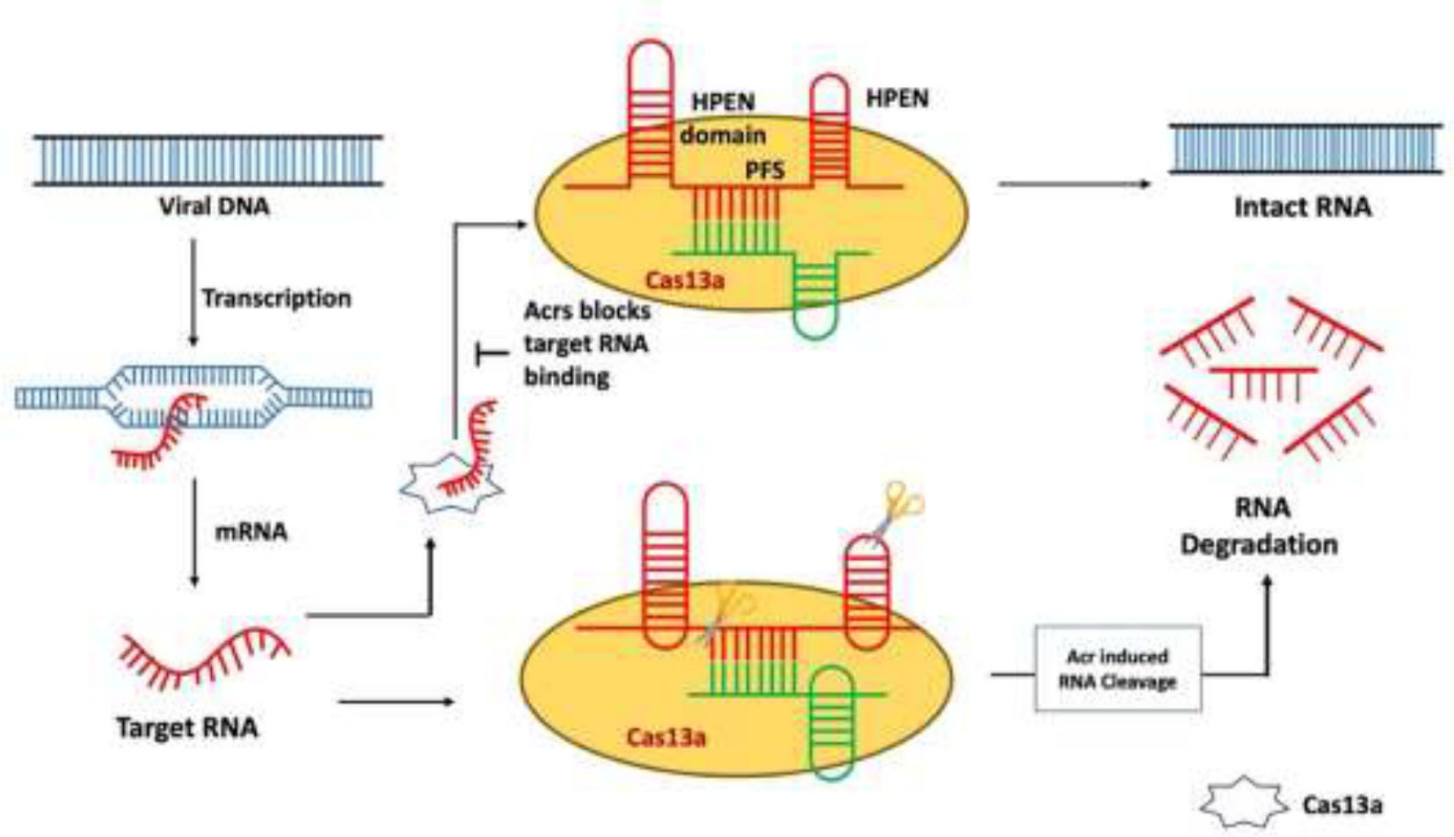
Mechanism of type VI anti-CRISPR defense strategy: AcrVIA1 inhibits Cas13a by contacting particular residues on the protein and gRNA, blocking the crRNA-exposed face of the nuclease from binding a complementary target RNA and activating Cas13a’s RNase activity. AcrVIA1 (or A2, A3, A4, A5, A6, and A7) prevents the HEPN domain from being cleaved by inhibiting the Cas13 protein (Jia and Patel, 2021).

After the type III CRISPR-Cas system type, VI is another mechanism in which viral RNA is targeted instead of DNA. Cas13a-mediated type VI CRISPR system is the simplest mechanism of nuclease as compared to other CRISPR systems. It only needs crRNA (gRNA + tracer RNA) and Cas13 protein (Cas13a/b/c/d) to occur. However, there are different Cas13 proteins such as C2c2 known as Cas13a, C2c6 known as Cas13b, C2c7 known as Cas13c, and proto-spacer flanking sequence (PFS)–independent Cas13d (**Table 1**) (Burmistrz et al., 2020). Considering the different features and functions of these Cas13 proteins, there are also some common factors like the presence of the HEPN domain. The HEPN domain is to indicate the cutting site for RNA targeting the Cas13 complex. Commonly, two HEPN domains are present in the Cas13 nucleolytic complex. The cleavage mechanism is followed up by PFSs, and these spacer sequences help in the recognition of the cleavage site (**Figure 5**) (Burmistrz et al., 2020). To capture this straightforward Cas system, Acr proteins target the most basic player of this mechanism, Cas13. The known seven Acrs for type VI CRISPR systems are denoted as AcrVIA1 to AcrVIA7 (Lin et al., 2020). Blocking the Cas13 protein AcrVIA1 (or A2, A3, A4, A5, A6, and A7) inhibits the cleavage at the HEPN domain.

## 6 Applications of anti-CRISPR

The versatile and effective use of the CRISPR-Cas system can be utilized for various applications, such as gene editing and chromatin imaging in eukaryotic cells (Jinek et al., 2012; Mali et al., 2013). Spatial and temporal investigations of chromatin dynamics are now possible because of the combination of live imaging of chromatin with programmable DNA binding proteins developed through genome-editing techniques (Fujimoto and Matsunaga, 2016). Acr proteins can be employed in novel ways to regulate CRISPR-Cas function because of their distinctive methods of action. Acrs can be used to enhance the activity of the CRISPR-Cas system in both prokaryotes and eukaryotes. The capability of numerous Acr proteins to directly interfere with CRISPR-Cas functions in heterologous hosts allows genetically encodable, post-translational regulation for technologies generated from CRISPR-Cas. Evaluation of the efficacy of CRISPR-Cas–based gene editing in target cells requires the detection of the CRISPR-Cas effector complex within biological materials. Acr-based biosensing technologies offer an alternative to antibodies for effector complex detection, identification, and quantification due to the high binding affinity of Acrs for effector complexes (Kaminski et al., 2021). The use of phage therapy and the use of bacterial viruses (phages) to treat bacterial infections are increasingly being studied as a potential replacement for antibiotics (Brives and Pourraz, 2020). The use of Acrs such as Acr IIA4, AcrVIA2, AcrVIA5, AcrIIA5, and AcrIIA2 is a proven method for minimizing the target’s effects of CRISPR-Cas tools in various hosts (Liang et al., 2020; Jia and Patel, 2021). Several research has provided evidence that Acrs such as AcrIIA4, AcrIIC1, and AcrIIC3 can be used to achieve cell-specific control of the gene-editing process carried out by CRISPR-Cas9 (Lee et al., 2019; Liang et al., 2020; Jia and Patel, 2021). Timed administration of AcrIIA2 and AcrIIA4 was shown to regulate CRISPR-Cas9 activity, decrease the cytotoxicity of human hematopoietic stem cells, and boost engraftment rates without impairing on-target genome editing (Jia and Patel, 2021). In *Saccharomyces cerevisiae*, it was discovered that the proteins AcrIIA2 and AcrIIA4 were able to deactivate Cas9, making them powerful gene drive inhibitors. Acr proteins have many potential applications, including the detection of persistent epigenetic modifications, the specific detection of CRISPR-Cas complexes, a promising tool for achieving CRISPR resistance in phage therapy, the restriction of editing activity to specific tissues or developmental stages, the enhancement of microbial gene-editing strategies, and the mitigation of toxic effects of genome editing.

## 7 Future challenges

Genome editing is currently being performed on more than 40 crop species across 25 countries to improve variety of traits, including agronomy, the quality of food and feed, and tolerance to abiotic stress in plants. There are currently six genome-edited agricultural traits available for commercialization; these traits may be found in soybeans, canola, rice, maize, mushrooms, and camelina (Menz et al., 2020; Pixley et al., 2022). There are numerous possible risks associated with genome-edited crops such as non-target mutations, breaking of natural reproductive barriers and intermediate transgenic elements (Pixley et al., 2022). The revolutionary discovery of Acr proteins has given us greater control over CRISPR-Cas editing (Pawluk et al., 2016a). There are a variety of potential applications for ACRs in the field of plant genome editing that can be tuned, such as (i) integration of cell-specific miRNA binding sites into ACRs to construct a cell type–specific Cas9-ON switch to alter plant genomes in a cell type–specific manner (Hoffmann et al., 2019) and (ii) enabling inducible plant genome editing by fusing a light-responsive domain with the CRISPR-Cas system to enable optogenetic control of the system (Bubeck et al., 2018; Liu et al., 2022). For instance, CASANOVA, which stands for “CRISPR-Cas9 activity switching *via* a novel optogenetic variant of AcrIIA4”, is a chimeric protein composed of AcrIIA4 fused to the LOV2 photosensor domain from the *Avena sativa* phototropin-1 protein. This domain is responsible for the protein’s ability to detect light (Yu and Marchisio, 2020). Acr can help with selective breeding, which could help plants and animals improve their valuable features. The Acr can be utilized as a helpful “off-switch” for the production of Cas9 activity in the gene therapy technique, as we know that phages are employed for gene therapy for the treatment of bacterial illness (Pawluk et al., 2016a). The type I-F Acr gene family has been shown to inhibit the type I-F CRISPR system in both *Pseudomonas aeruginosa* and *Pectobacterium atrosepticum*, and type II-A Acr proteins can block the expression of Cas9 protein in most studies (Marino et al., 2018; Mir et al., 2018; Bhoobalan-Chitty et al., 2019; Athukoralage et al., 2020). Acr protein not only inhibits some Cas proteins during the editing process but also allows us to fix mistakes or off-target mutations afterward while also partially blocking editing at the targeted location (Athukoralage et al., 2020). Numerous studies have shown that Acr proteins have a wide spectrum of functional activity, allowing researchers to use them to modify the insertion, deletion, silence, and single-letter fixation of any characteristic. Identifying the most prevalent architectural or dynamic aspects of Acr-Cas interactions is crucial for predicting inhibitory consequences with novel or planned Acrs; hence, studying these biophysical principles is of paramount importance. Because of their high affinity and specificity for CRISPR-Cas systems, Acrs have the potential to cure a wide range of diseases, including those caused by multidrug-resistant bacteria; secondary bacterial infections associated with COVID-19 and SARS-CoV; disorders associated with defective genes like Alzheimer, Parkinson, and Huntington’s diseases; diseases transmitted by insects; and viral diseases through regulated genome editing. At this point of time, the Acr protein–based treatments and the role that they play in plant research are in its nascent stage. Exploring the biophysical principles important for Acr function is essential to pinpointing the most common architectural or dynamic features of Acr-Cas interactions, which can be used to predict future inhibitory outcomes with novel or designed Acrs.

## Author contributions

NC, DT, RV, and B-HJ designed the idea and write the first draft of the manuscript; VY, KY, RG, ND, MHA, MSA, and TG reviewed the manuscript; MC, DT, LE, AG, and NC prepared the final draft; RV and B-HJ finalized and submitted the manuscript. All authors contributed to the article and approved the submitted version.
